# Obesity, Independent of p53 Gene Dosage, Promotes Mammary Tumor Progression and Upregulates the p53 Regulator MicroRNA-504

**DOI:** 10.1371/journal.pone.0068089

**Published:** 2013-06-28

**Authors:** Nikki A. Ford, Sarah M. Dunlap, Karrie E. Wheatley, Stephen D. Hursting

**Affiliations:** 1 Department of Nutritional Sciences University of Texas at Austin, Austin, Texas, United States of America; 2 Department of Molecular Carcinogenesis, University of Texas M.D. Anderson Cancer Center, Smithville, Texas, United States of America; Roswell Park Cancer Institute, United States of America

## Abstract

Obesity, prevalent in >35% of US women, is an established risk and progression factor for postmenopausal breast cancer, and strategies to break the obesity-breast cancer link are urgently needed. Approximately 30% of breast cancers carry p53 tumor suppressor gene alterations; however, the effects of obesity on breast cancer progression in relation to p53 gene dosage are unclear. Using murine models of postmenopausal breast cancer, we characterized the interactive effects of diet-induced obesity (DIO) and p53 gene dosage on mammary tumor growth and associated p53-related regulatory mechanisms. Ovariectomized C57BL/6 mice were randomly assigned to receive a DIO or control diet, and (at 10 weeks) orthotopic injection of MMTV-Wnt-1 p53^+/−^ or MMTV-Wnt-1 p53^+/+^ mammary tumor cells (n = 20 mice per diet and genotype group). DIO and control diets produced distinct phenotypes (mean percent body fat at 10 weeks: 57% and 39%, respectively, *P*<0.001). Regardless of phenotype, time to first palpable tumor was 57% less for Wnt-1 p53^+/−^ than Wnt-1 p53^+/+^ tumors. Regardless of tumoral p53 genotype, DIO (relative to control) increased tumor burden, tumor cell proliferation (Ki-67), severity of tumor pathology, local tissue invasion, epithelial-to-mesenchymal transition (EMT) programming, and tumoral microRNA-504 (a negative regulator of p53) expression; and suppressed p53, p21, and estrogen receptor-alpha protein expression. These findings in murine models of postmenopausal breast cancer suggest that obesity may augment procancer effects related to p53 gene alterations. Furthermore, microRNA-504, an obesity-responsive negative regulator of p53 and putative EMT regulator, may represent a novel molecular target for breaking the obesity-breast cancer link.

## Introduction

Breast cancer is the most commonly diagnosed noncutaneous cancer among women in the United States [1]. Epidemiologic studies have identified obesity as an important modifiable risk factor for postmenopausal breast cancer. Obese postmenopausal women typically have altered serum levels of metabolic hormones, such as insulin, insulin-like growth factor (IGF)-1, leptin, resistin and adiponectin, and are at increased risk for developing breast cancer [2], [3], [4]. Obese women also generally have poorer prognosis than their lean counterparts if they develop breast cancer [5]. Since the prevalence of obesity in US women exceeds 35% [6] and since postmenopausal breast cancer rates are increasing [7], new molecular targets and strategies for offsetting the effects of obesity on postmenopausal breast cancer are urgently needed.

The p53 tumor suppressor gene is altered in approximately 30% of all breast tumors, with the highest functional loss (>80%) in basal-like breast tumors [8]. However, the effects of obesity on breast cancer development and progression are not well established in relation to p53 gene dosage. The fate of a cell in response to injury or mutation is largely determined by the ability of p53 to inhibit cell cycle progression and initiate repair or cell death [9]. Negative regulators of p53 include the p53 acetylator Sirtuin 1 (SIRT1) [10], the ubiquitin ligase mouse double minute 2 (MDM2) [11], microRNA (miR)-125b [12] and miR-504 [13]. The impact of obesity on SIRT1 [14] and MDM2 [15] is well established in several tissues. However, the effect of obesity on miR-125b and miR-504, which negatively regulate p53 through direct binding in the 3′ untranslated region of the gene, resulting in decreased p53 protein translation (7,8), is unclear.

The growth and progression of breast cancer is also impacted by crosstalk between p53, estrogen receptor-alpha (ERα) and the epithelial-to-mesenchymal transition (EMT) program [16], although the effect of obesity on this crosstalk is poorly characterized. ERα is an established breast cancer-related regulator of p53 [17], [18], [19], and in turn, p53 regulates ERα through a feed-forward mechanism [20], [21], [22]. EMT is a key developmental program that is often re-initiated during cancer progression, leading to increased invasion and metastasis [23] and progressive loss of epithelial markers (eg, E-cadherin, keratin 8) and gain of mesenchymal markers (eg, slug, snail, zeb1, vimentin) [24]. Interactions between obesity, ERα, p53 and EMT are highly plausible because: 1) obesity increases mammary aromatase expression and serum estradiol levels [25]; 2) obesity upregulates EMT in MMTV-Wnt-1 basal-like mammary tumors and Met-1 luminal-type mammary tumors expressing wild-type p53 [26], [27]; 3) the loss of p53 in human breast cancer cells results in EMT-associated stem cell-like features [28]; and 4) p53 suppresses cell invasion by initiating the degradation of slug and increasing E-cadherin expression [29], and slug, in turn, can repress p53-mediated apoptosis [30].

In the current study, we modeled postmenopausal basal-like breast cancer in the context of different p53 gene dosage by injecting MMTV-Wnt-1 mammary tumor cells that were either p53^+/+^ or p53^+/−^ into the mammary fat pads of ovariectomized female C57BL/6 mice. Using these Wnt-1 p53^+/+^ and Wnt-1 p53^+/−^ mammary tumor models, we characterized the effects of diet-induced obesity (DIO) and p53 expression on postmenopausal mammary tumor growth. We also evaluated p53-related regulatory mechanisms to identify molecular targets for breaking the obesity-breast cancer link.

## Results

### DIO and Control Diet Regimens Induced Distinct Phenotypes

**Figure 1:**
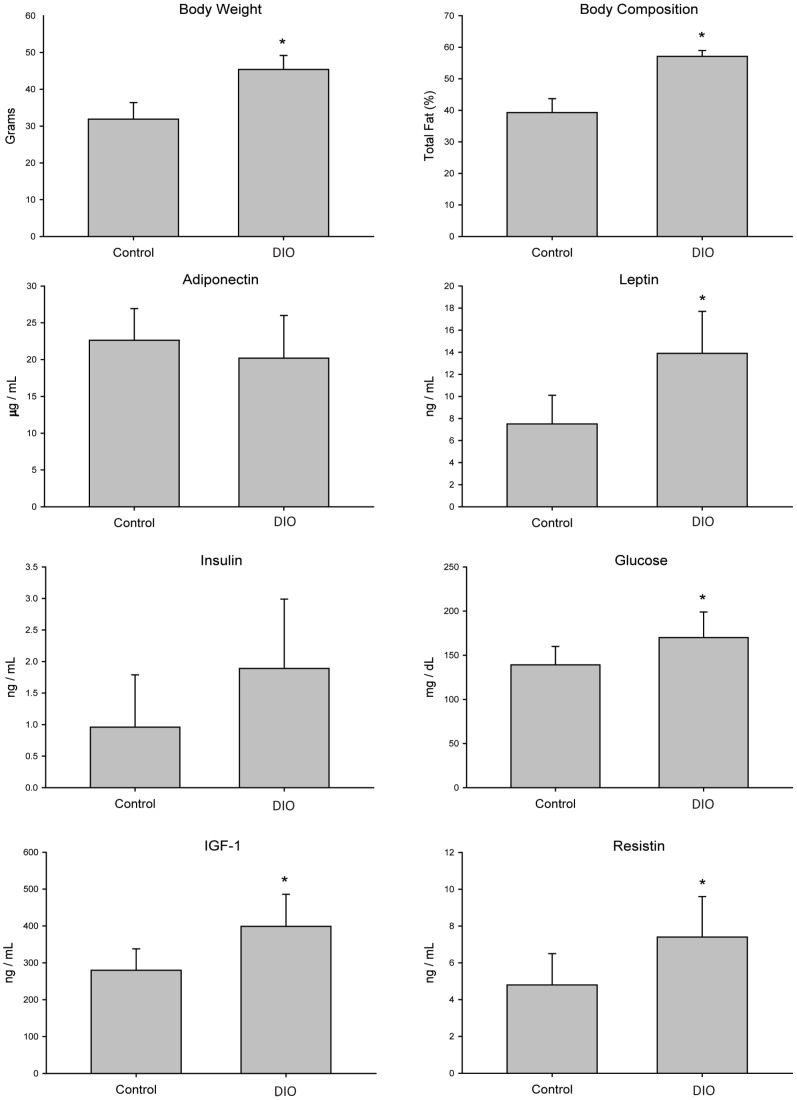
10 weeks of feeding DIO and control diets impacts body size and metabolic phenotypes in ovariectomized C57BL/6 mice (n = 40 mice per diet). Significant differences are indicated by an asterisk; *P*≤0.05.

DIO and control diets effectively generated two different body size and metabolic phenotypes by 10 weeks in ovariectomized C57BL/6 mice (n = 40 mice per diet): DIO (45±3.8 g; 57±1.9% body fat) and control (32±4.5 g; 39±4.4% body fat) (P<0.001 each parameter) (Figure 1). Consistent with previous findings [31], DIO, relative to control, diet significantly increased serum IGF-1 (410±39 ng/mL versus 310±18 ng/mL; P = 0.019), serum leptin (12±0.6 ng/mL versus 6.4±0.5 ng/mL; P<0.001), blood glucose (180±8.6 mg/dL versus 160±8.5 mg/dL; P = 0.048) and serum resistin levels (5.1±2.1 ng/mL versus 7.6±2.3 ng/mL; P = 0.044). DIO did not significantly raise serum insulin levels (1.7±0.4 ng/mL) compared to control mice (0.9 ng/mL ±0.3 ng/mL; P = 0.067) or significantly reduce serum adiponectin (17±1.4 µg/mL) relative to control mice (23±1.1 µg/mL; P = 0.080) (Figure 1).

### Characterization of Wnt-1 Tumor Cell Suspensions

**Figure 2:**
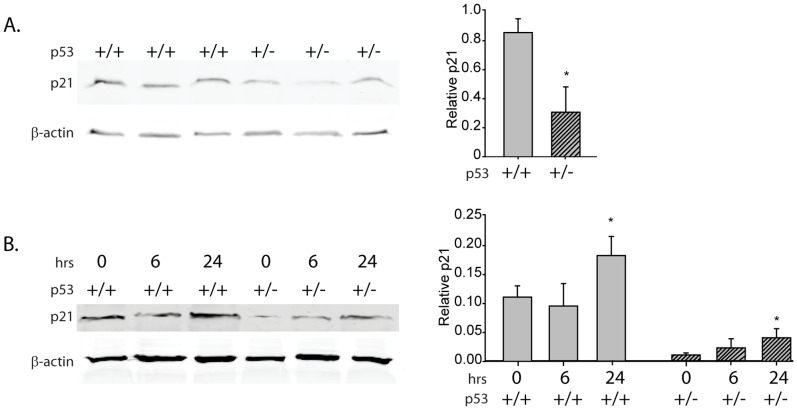
p21 protein in Wnt-1 p53^+/+^ and Wnt-1 p53^+/−^ tumor suspensions. A, p21 protein by immunoblot in Wnt-1 p53^+/+^ and Wnt-1 p53^+/−^ tumor cell suspensions (approximately 1 million cells) and B, in response to UVC DNA damage after 0, 6 and 24 hours. Significant differences in protein levels are indicated by an asterisk on the densitometry plots; P≤0.05.

We previously demonstrated p53 heterozygosity and greater induction of p53 expression by the DNA damaging agent doxorubicin in Wnt-1 p53^+/−^ tumor cells relative to Wnt-1 p53^+/−^ tumor cells [21], [32]. In the present study, protein levels of a key p53 target, p21, were also markedly reduced in the Wnt-1 p53+/− tumor cells relative to the Wnt-1 p53+/+ tumor cells (Figure 2A and 2B). Furthermore, enhanced protein expression of p21 occurred in response to UVC-induced DNA damage in Wnt-1 p53+/+ and p53+/− tumor cells (Figure 2B).

### Loss of p53 and/or a DIO Regimen Increased Wnt-1 Mammary Tumor Growth and Modulated Tumor Pathology

**Figure 3:**
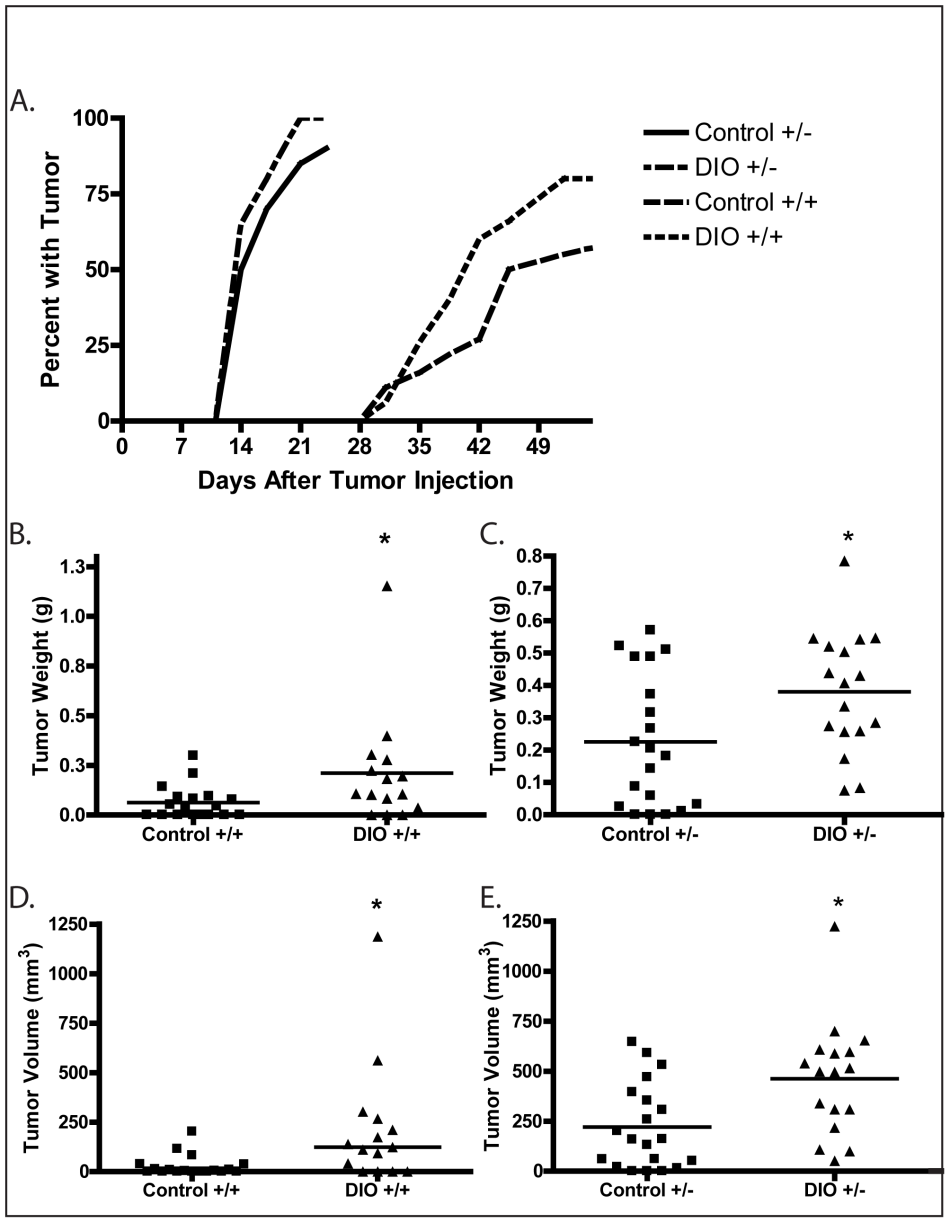
Effect of p53 gene dosage and a DIO regimen versus a control diet on Wnt-1 mammary tumor growth. A, the percentage of mice with palpable Wnt-1 mammary tumors after tumor cell injection in response to a control diet or DIO regimen, (n = 20 per group). Wnt-1 p53^+/+^ and Wnt-1 p53^+/−^ final tumor weights are shown in B and C, respectively, and Wnt-1 p53^+/+^ and Wnt-1 p53^+/−^ final tumor volumes are shown in D and E, respectively (means indicated by horizontal lines). Significant differences are indicated by an asterisk; *P*≤0.05.

Wnt-1 p53^+/−^ or Wnt-1 p53^+/+^ mammary tumor cells were injected into the fourth mammary fat pads of the mice after 10 weeks of DIO or control diet regimen (n = 20 mice per diet and genotype). The resulting Wnt-1 p53^+/−^ mammary tumors were palpable significantly earlier than Wnt-1 p53^+/+^ tumors (13 days versus 30 days post-injection; P<0.001). At least one tumor reached 1.0 cm in length or width by 23 days post-injection of Wnt-1 p53^+/−^ tumor cells and 52 days post-injection of Wnt-1 p53^+/+^ tumor cells (Figure 3A). At these respective times, 100% (20/20) of DIO and 90% (18/20) of control mice that had been injected with Wnt-1 p53^+/−^ mammary tumor cells had palpable tumors, and 80% (16/20) of DIO and 55% (11/20) of control mice had palpable Wnt-1 p53^+/+^ tumors. At final measurement, DIO mice, relative to control mice, had significantly increased Wnt-1 p53^+/+^ tumor weight (P = 0.044; Figure 3B) and volume (P = 0.010; Figure 3D), and significantly increased Wnt-1 p53^+/−^ tumor weight (P = 0.022; Figure 3C) and volume (P = 0.005; Figure 3E).

**Figure 4:**
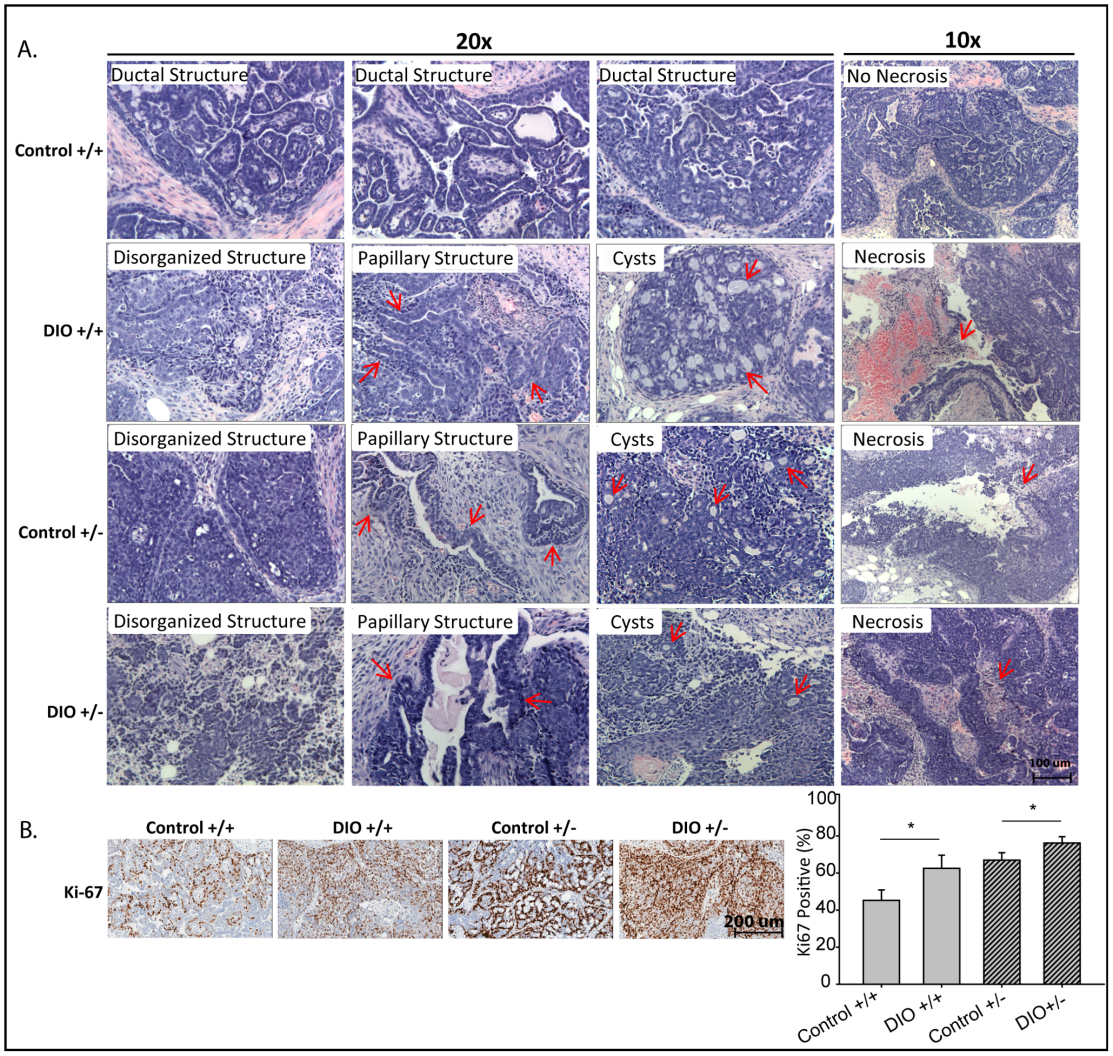
Effect of p53 gene dosage and a DIO regimen versus a control diet on Wnt-1 mammary tumor pathology and proliferation. A, representative photomicrographs of hematoxylin- and eosin-stained sections of Wnt-1 p53^+/+^ and Wnt-1 p53^+/−^ tumors from mice fed a control diet or DIO regimen (n = 11–20 mice/group; 10–20×). Each tumor-bearing mouse developed a single tumor. Arrows point to indicated pathological structures. B, representative photomicrographs of immunohistochemical staining of tumors for Ki-67 (20×) and a bar graph presenting the Aperio image quantitation, means ± SD, (n = 5 per group). Significant differences are indicated by an asterisk; *P*≤0.05.

Histopathological examination revealed that Wnt-1 p53^+/+^ mammary tumors from control mice were well-differentiated ductal adenocarcinomas with clearly defined margins and no central necrosis (Figure 4A). In contrast, Wnt-1 p53^+/+^ mammary tumors from DIO mice and Wnt-1 p53^+/−^ mammary tumors from control mice or DIO mice were all poorly differentiated ductal adenocarcinomas with disorganized ductal structures, papillary and cystic structures and wide spread central necrosis. DIO, relative to control, significantly increased cell proliferation, as assessed by immunohistochemical staining against Ki-67, in Wnt-1 p53^+/+^ tumors (63±7.1% versus 45±5.7% positive cells/field, respectively; *n* = 5/group; P = 0.030) and Wnt-1 p53^+/−^ tumors (76±3.4% versus 67±4.0% positive cells/field; n = 5/group; P = 0.038; Figure 4B).

### DIO Suppressed p53 Signaling and Increased miR-504 Expression in Wnt-1 Mammary Tumors

**Figure 5:**
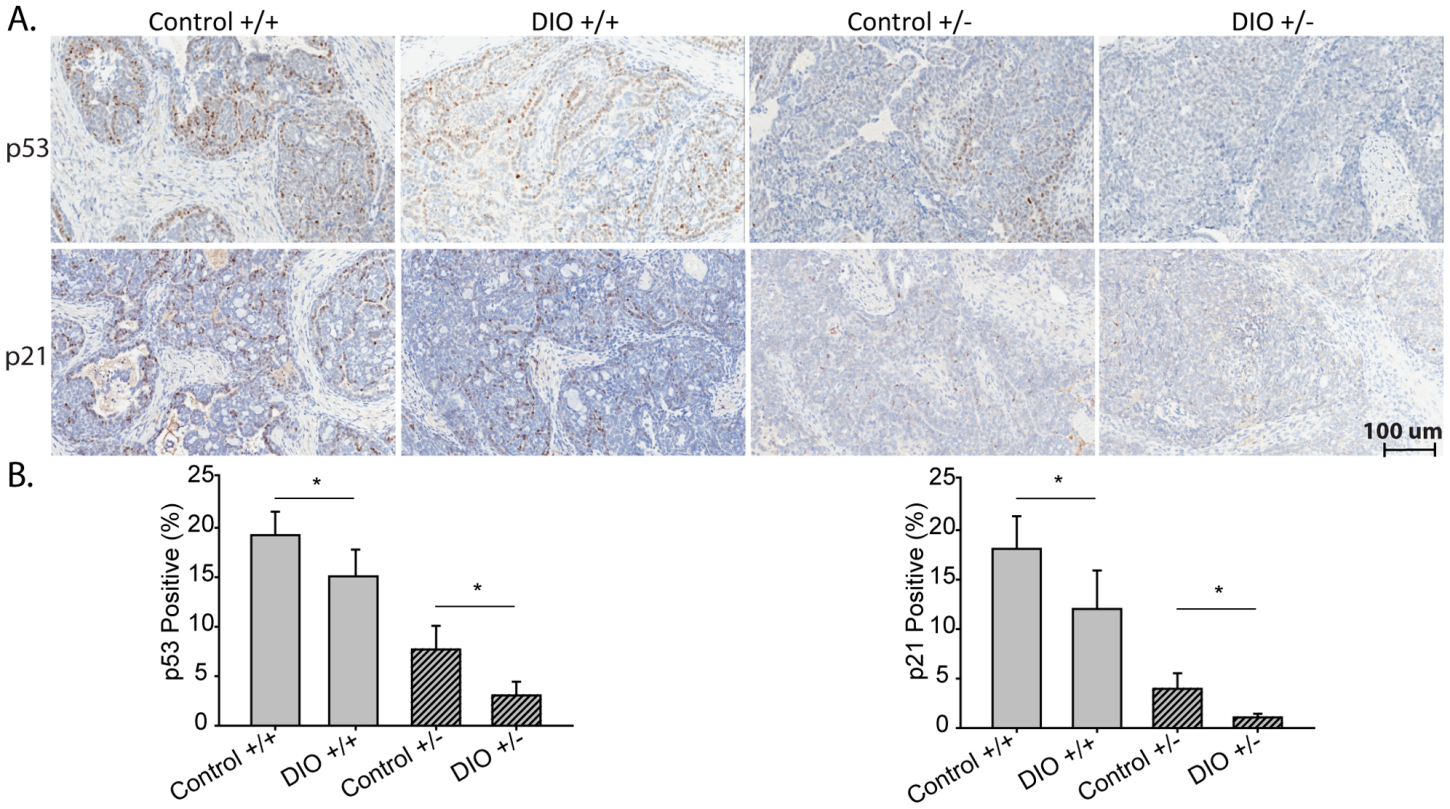
The effect of diet on p53 signaling in Wnt-1 p53^+/+^ and Wnt-1 p53^+/−^ tumors. A, Representative photomicrographs of immunohistochemical staining of Wnt-1 p53^+/+^ or Wnt-1 p53^+/−^ mammary tumors for p53 and p21 (20×) with B, bar graphs presenting Aperio quantitation from mice fed a control diet or DIO regimen, means ± SD, (n = 5 per group). Significant differences are indicated by an asterisk; *P*≤0.05. p21 IHC data was natural log transformed to meet statistical test assumptions.

**Figure 6:**
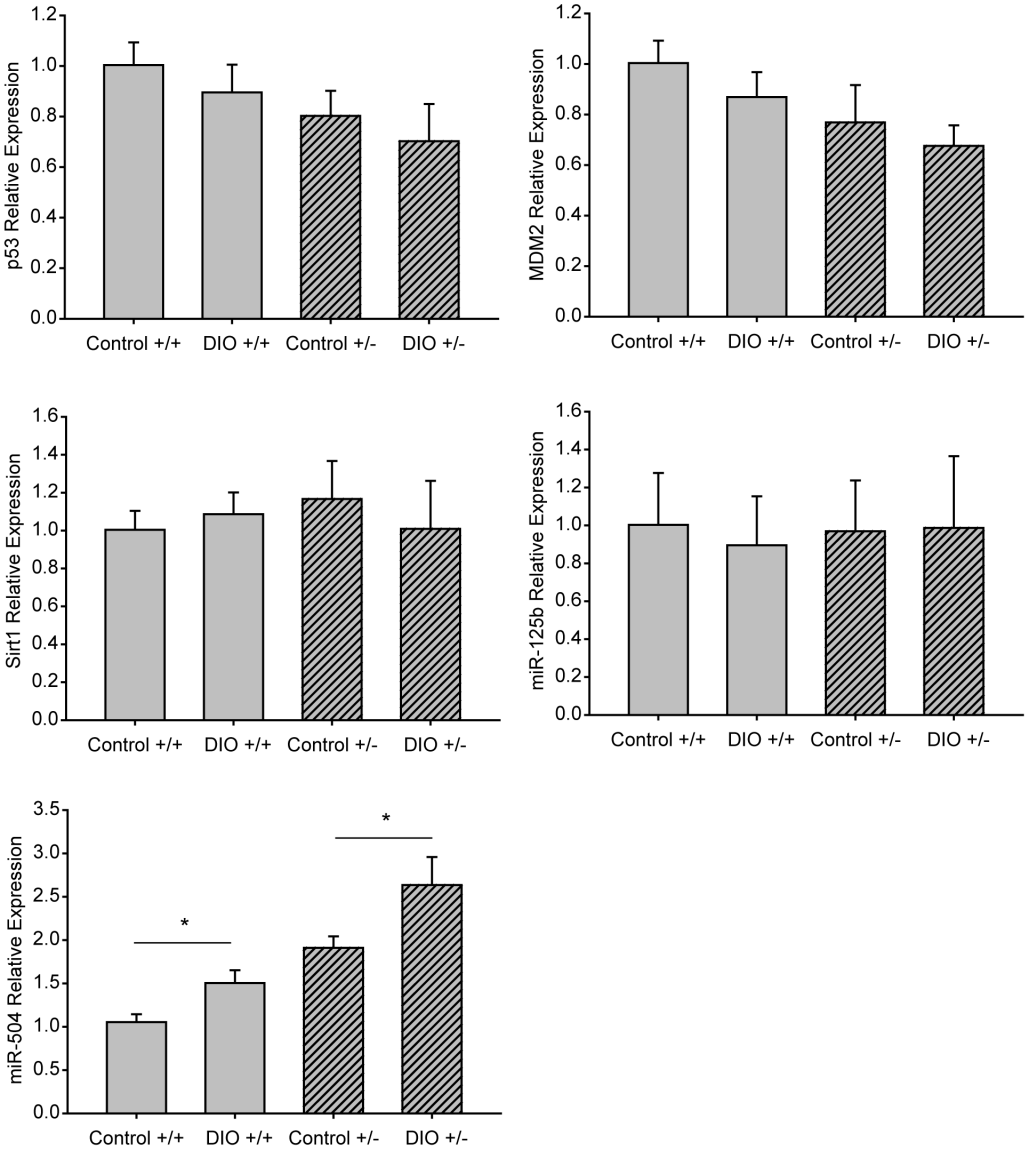
Effect of p53 gene dosage and a DIO regimen versus a control diet on Wnt-1 mammary tumor gene expression of p53 and its regulators. mRNA expression, measured by quantitative real-time PCR, of p53, mouse double minute (MDM2), Sirtuin (Sirt)1, microRNA (miR)-125b and miR-504 (n = 5 per gene per group; 3 replicates) in Wnt-1 p53^+/+^ and Wnt-1 p53^+/−^ tumor tissue from DIO or control mice. Data are presented as gene expression relative to that in Wnt-1 p53^+/+^ tumors from control mice (means ± SD). Significant differences are indicated by an asterisk; *P*≤0.05. miR-125b data was natural log transformed to meet statistical test assumptions.

p53 protein expression, determined by immunhistochemistry (Figure 5), was significantly lower in Wnt-1 p53^+/+^ tumors from DIO mice, relative to control mice (16% reduction; n = 5/group; P = 0.047), and in Wnt-1 p53^+/−^ tumors from DIO mice, relative to control mice (60% reduction; n = 5/group; P = 0.020). DIO, relative to control, also significantly reduced the expression of p21, a downstream target of p53 and regulator of cell cycle progression, by 33% in Wnt-1 p53^+/+^ tumors (P = 0.050) and 73% in Wnt-1 p53^+/−^ tumors (P = 0.003). Statistically significant DIO-dependent effects on p53 gene expression were not detected in either genotype (Figure 6).

The mRNA expression of Mdm2, Sirt1 and miR-125b, each of which negatively regulates p53 activity, was not modulated by DIO, relative to control, in Wnt-1 p53^+/+^ or Wnt-1 p53^+/−^ mammary tumor tissue (Figure 6). In contrast, DIO, relative to control, significantly increased expression of miR-504, also a negative regulator of p53, in Wnt-1 p53^+/+^ tumor tissue (P = 0.013) and Wnt-1 p53^+/−^ tumor tissue (P = 0.032, Figure 6). Expression of miR-504 in the tumor cell suspensions (same batch used for the tumor transplant study) was also significantly increased in the Wnt-1 p53^+/−^ cells compared with Wnt-1 p53^+/+^ cells (1.91+/−0.11 versus 1.05+/−0.12 relative expression units; P = 0.002).

### DIO Promoted Invasion and EMT in Wnt-1 Mammary Tumors

**Figure 7:**
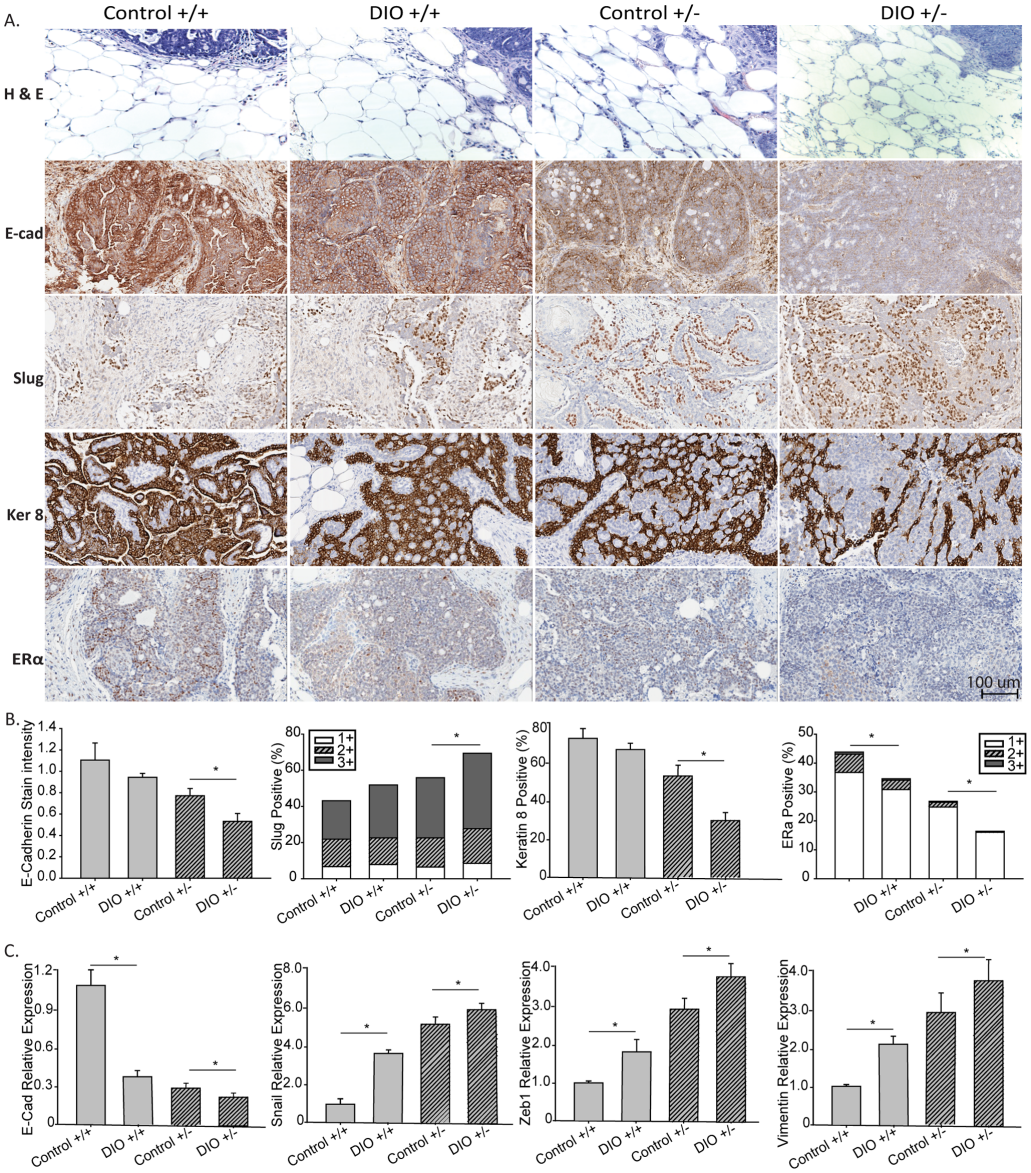
Effect of p53 gene dosage and a DIO regimen versus a control diet on Wnt-1 mammary tumor on invasive markers and ERα expression. A, representative hematoxylin and eosin images (n = 11–20; 10–20×) of Wnt-1 p53^+/+^ and Wnt-1 p53^+/−^ tumors and surrounding mammary fat pads from mice fed a control diet or DIO regimen. Representative micrographs of immunohistochemical staining of tumors for e-cadherin, slug, keratin 8 and ERα (20×),B, bar graphs presenting Aperio quantification, and C, bar graphs depicting gene expression of EMT markers, means ± SD, (n = 5 per group). Significant differences are indicated by an asterisk; *P*≤0.05. Positive staining for ERα and slug was further stratified by 1+, low; 2+, moderate; and 3+, intense staining.

Tumor sections were examined histologically and immunohistochemically for evidence of obesity-related invasion and induction of EMT, in the context of differential p53 gene dosage (Figure 7). Wnt-1 p53^+/+^ mammary tumors from control mice displayed a clearly defined, encapsulated border between tumor and mammary fat pad with minimal adipocyte infiltration (Figure 7A, hematoxylin- and eosin-stained sections). In contrast, Wnt-1 p53^+/+^ tumors from DIO mice, Wnt-1 p53^+/−^ tumors from control mice, and to a greater extent, Wnt-1 p53^+/−^ tumors from DIO mice, lacked defined borders, showed marked infiltration to the adjacent mammary fat pad, and adipocyte infiltration into the tumors.

Invasion of tumor cells into surrounding tissue often requires induction of EMT, as evidenced by loss of E-cadherin and keratins and/or increased expression of mesenchymal markers such as slug [24], [33]. DIO significantly reduced E-cadherin expression in Wnt-1 p53^+/−^ tumors (P = 0.005), reduced keratin 8 expression (P = 0.009) and increased total slug expression (P = 0.010), relative to control mice (Figure 7). DIO, relative to control, also decreased E-cadherin and keratin 8 expression and increased total slug expression in Wnt-1 p53^+/+^ tumor tissue, although the differences did not reach statistical significance (P = 0.080, P = 0.240 and P = 0.120, respectively). The distribution of slug staining intensity levels was not significantly altered by DIO in either tumor genotype (Figure 7B). mRNA expression of key EMT genes was also modified by DIO (Figure 6C). Specifically, DIO significantly reduced E-cadherin expression in Wnt-1 p53^+/+^ and Wnt-1 p53^+/−^ tumors (P<0.001 and P = 0.031, respectively). DIO also upregulated the mRNA expression of snail, zeb1 and vimentin in Wnt-1 p53^+/+^ (P<0.001, P = 0.028 and P = 0.019, respectively) and Wnt-1 p53^+/−^ tumors (P = 0.043, P = 0.027 and P = 0.049, respectively).

### DIO Decreased ERα Expression in Wnt-1 Mammary Tumors

Based on immunohistochemical staining (Figure 7B), DIO relative to control significantly reduced ERα expression in Wnt-1 p53^+/+^ (P = 0.006) and Wnt-1 p53^+/−^ (P = 0.047) mammary tumors. The distribution of ERα staining intensity (as a percentage of total) was not altered by DIO in either tumor genotype (Figure 7B).

## Discussion

Using Wnt-1 p53^+/+^ and Wnt-1 p53^+/−^ mouse models of postmenopausal basal-like breast cancer, we provide evidence that a DIO regimen (relative to control diet and regardless of tumoral p53 genotype): i) promotes mammary tumor progression, more severe tumor pathology, EMT, and loss of ERα protein expression; and ii) suppresses tumoral p53 and p21 protein expression in association with increased expression of the negative regulator of p53, miR-504.

The enhancing effects of reduced expression or mutation of p53 on breast cancer progression are well documented, and we [26], [34], [35], [36] and others [3], [37], [38] have demonstrated that calorie restriction and DIO differentially impact mammary tumor development and progression. Calorie restriction, an antiobesity diet regimen, increases longevity and inhibits many types of cancer in numerous animal models [39]. We previously reported that in p53-deficient and wild-type mice, calorie restriction inhibits spontaneous lymphomas and sarcomas in a p53-independent fashion [40], [41]. However, the effect of DIO on cancer in the context of reduced p53 expression has, to our knowledge, not yet been published. Herein, we report that, regardless of body size phenotype, the first palpable tumor appeared 57% earlier for Wnt-1 p53^+/−^ than Wnt-1 p53^+/+^ tumors, consistent with the tumor-enhancing effects of decreased p53 gene dosage. Furthermore, we found that DIO significantly increases mammary tumor growth, induces a more aggressive pathology, and represses tumoral p53 and p21 protein expression in both Wnt-1 p53^+/+^ and Wnt-1 p53^+/−^ tumors (Figure 5). Overall, we found that DIO decreases p53 protein expression, which appears to promote a pathology and proliferative environment in Wnt-1 p53^+/+^ tumors more reflective of Wnt-1 p53^+/−^ tumors from control mice than Wnt-1 p53^+/+^ tumors from control mice. Consistent with our observation that DIO decreases tumoral p53 levels, Park et al. has demonstrated that a high-fat, high-calorie diet significantly reduced p53 levels in murine colon tumors [42]. Thus, the procancer effects of DIO may, in part, be attributed to constraint of p53 tumor suppressor function.

In this mammary tumor model no metastases were detected in the liver or lungs. The primary tumors grew very quickly, which may not have allowed sufficient time for metastases to develop and become established in distal sites prior to termination of the study. Previously, we demonstrated that Wnt-1 p53^+/+^ tumor cells contain a population of cells that has a mesenchymal phenotype with stem cell-like characteristics [26]. To further address whether DIO impacts metastases, studies are underway using classic metastasis model development approaches, including removal of the primary tumor, and tail vein, cardiac and splenic injections of tumor cells.

In the present study in Wnt-1 tumors differing in p53 gene dosage, well-established (Mdm2, Sirt1 and miR-125b) and emerging (miR-504) negative regulators of p53 [10], [11], [12], [13] were investigated in response to DIO versus control diet (Figure 6). DIO did not appear to target Mdm2, Sirt1 or miR-125b. However, tumoral miR-504 and p53 levels were inversely associated, suggesting that the observed DIO upregulation of miR-504 expression contributes to the reduced p53 protein (but not mRNA) expression. It is plausible that the observed post-transcriptional, but not transcriptional, decrease in p53 levels are under microRNA regulation, since microRNA’s typically repress protein translation of the target mRNA [12]. Regulation of miR-504 has not yet been well characterized, but growth factors or hormones modulated by obesity may play a role. We found that DIO elevated serum leptin and IGF-1. Leptin suppresses p53 expression in human LNCaP prostate cancer cells and human ZR-75-1 breast cancer cells [43], [44]. Additionally, serum leptin and insulin levels suppress p53 in diabetic rats resulting in enhanced bladder, liver and colon cancer [45]. IGF-1 binding to its receptor activates the Akt/mammalian target of rapamycin pathway [46] and suppresses AMP kinase [47], both of which regulate p53 activity. Future studies on the links between obesity-related hormones, miR504 and p53 are warranted.

Another possible role of miR-504 in DIO-associated mammary tumor progression involves the putative interaction between miR-504, p53 and EMT. In human glioblastomas, miR-504 expression correlates with the expression of several EMT- or stem cell-related markers [48]. We demonstrated that DIO fosters local mammary fat pad invasion and promotes the molecular hallmarks of EMT (Figure 7), potentially through abrogation of p53 function [49]. Functional p53 represses the expression of CD44 and therefore the gain of stem cell-like properties typical of EMT [50], [51]. In contrast, with loss of p53 function, transcriptional changes occur in several EMT-related markers in association with enhanced tumor progression and metastasis of human breast cancer xenografts [28]. Additionally, using silencing technology, Morel, et al. demonstrated that human mammary epithelial cells express mesenchymal markers and progress through malignant transformation when deprived of p53 [52]. Chang, et al and Kim, et al independently suggest p53-driven EMT programming depends upon the presence of microRNAs that are activated with p53 loss and in turn activate EMT [53], [54].

Another novel finding from our study is that DIO decreases p53 and ERα protein expression in association with enhanced Wnt-1 mammary tumor progression and irrespective of tumoral p53 genotype. Estrogen receptor modulators effectively reduce development and reoccurrence of ERα-positive breast cancers, and loss of tumoral ERα expression generally precludes estrogen-targeted therapies [55]. Additionally, more aggressive breast cancers such as basal-like, triple negative and claudin-low subtypes, present as ERα-negative tumors [56]. Therefore preventing the loss of ERα positivity may improve therapeutic outcomes in patients. Our observed suppression of tumoral ERα expression by the DIO regimen may be attributed to loss of p53 expression (Figure 7). Studies in MMTV-Wnt-1 transgenic mice demonstrated that loss of p53 decreases the anticancer response to tamoxifen and promotes the development of ERα-negative mammary tumors [21], [22]. Moreover, in human MCF-7 and murine Wnt-1 mammary tumor cells, p53 regulates ERα transcription through direct binding of the ER promoter [20], [21].

In summary, using Wnt-1 p53^+/+^ and Wnt-1 p53^+/−^ mammary tumor models, we report that DIO, irrespective of p53 gene dosage, promotes postmenopausal mammary cancer. Specifically, DIO, relative to control, increases mammary tumor progression, local tissue invasion, and EMT programming, and suppresses protein expression of p53, p21, and ERα, possibly through elevated miR-504 expression. These findings suggest that obesity may mimic or augment procancer effects related to p53 gene alterations. Furthermore, miR-504, an obesity-responsive negative regulator of p53 (and hence p21 and ERα) and putative regulator of EMT, may represent a novel molecular target for breaking the obesity-breast cancer link.

## Materials and Methods

### Ethics Statement

The animal study was approved by the University of Texas at Austin Institutional Animal Care and Use Committee.

### Mice, Diets and Study Design

Six-week old ovariectomized female C57BL/6 mice (n = 80) were purchased from Taconic Farms, Inc. (Germantown, NY), individually housed on a 12-hour light/dark cycle, and placed on a chow diet (Harlan Diets, Madison, WI) for one week. Mice were then randomly assigned (n = 40 per group) to receive either a control diet (modified AIN-76A diet with 10 kcal% fat; Research Diets, New Brunswick, NJ, #D12450B) or a DIO diet (modified AIN-76A with 60 kcal% fat; Research Diets #D12492), fed ad libitum. Body weights and caloric intake were analyzed weekly. After 10 weeks, all mice within a diet group were randomized to be orthotopically injected with 5×10^4^ Wnt-1 p53^+/+^ or Wnt-1 p53^+/−^ (n = 20 per diet group) murine mammary tumor cells into the 4th mammary fat pad, as previously described for Wnt-1 p53^+/+^ cells [57]. Tumor diameters were measured in two dimensions (d_1_ and d_2_) twice weekly with electronic skin fold calipers, and tumor volume was approximated using the formula 4/3π(d_1_/2)^2^(d_2_/2), where d_1_< d_2_ [57]. Each tumor-bearing mouse developed a single tumor. When the first tumor of a particular p53 genotype reached 1.0 cm in either length or width, all mice with that genotype were fasted for 10 hours and anesthetized with isoflurane followed by cardiac puncture for blood collection. Fasting blood glucose was immediately measured using an Ascencia Elite XL 3901G glucose analyzer (Bayer Corporation, Mishawaka, IN). Mice were then killed by cervical dislocation, and tumors were excised and either fixed in 10% neutral-buffered formalin or flash frozen in liquid nitrogen and stored at -80°C until further analysis. Blood samples were allowed to coagulate for 30 minutes at room temperature, then centrifuged at 10,000×*g* for 5 min to obtain serum aliquots that were stored at −80°C until assayed.

### Wnt-1 Tumor Cell Suspension

Wnt-1 mammary tumor cells with wild-type p53 (p53^+/+^) or heterozygous p53-deficiency (p53^+/−^) were obtained from spontaneous mammary tumors from MMTV-Wnt-1 transgenic mice crossed with p53^+/+^ or p53^+/−^ mice (B6;129-Trp53tm1Brd N12 homozygous and heterozygous and back-crossed to C57BL/6 for 10 generations). Tumors were collected aseptically from donor mice using blunt dissection, trimmed of extraneous tissues, mechanically dissociated by mincing and passage through a 40-micron mesh sterile screen, and suspended in serum-free RPMI 1640 (Gibco Laboratory, Grand Island, NY). Cells were further dissociated by serial passage through a syringe with an 18-gauge needle. Viable cells from multiple tumors were suspended in RPMI-1640 medium (Gibco Laboratory, Grand Island, NY) and quantitated using a hemacytometer prior to injection into the 4^th^ mammary fat pad.

### Body Composition and Serum Hormones

Percent body fat was assessed on each mouse carcass by dual-energy X-ray absorptiometry (GE Lunar PIXImus II, Madison, WI). The head of each mouse was eliminated from the scan by using the region of interest exclusion option provided in the software. Nine randomly selected serum samples per diet and genotype group were assayed using mouse adipokine LINCOplex® Multiplex Assays (Millipore, Inc., Billerica, MA), according to the manufacturer’s instructions on a BioRad Bioplex 200 analysis system (Biorad, Inc. Hercules, CA) to measure insulin, leptin, resistin and adiponectin. Total serum IGF-1 was measured using Mouse/Rat IGF-1 Quantikine ELISA Kit (R&D System, Inc., Minneapolis, MN) per manufacter’s instructions.

### Histopathology and Immunohistochemical Staining

Paraffin embedded tumor tissue (5 tumors per diet and genotype group, randomly selected) was cut into 4-µm thick sections for either hematoxylin and eosin staining or immunohistochemical analysis. Wnt1 mammary tumor development was assessed histologically (in a blinded fashion by the veterinary pathologist), by identification of ductal structure, papillary or cystic structures and necrosis. For immunohistochemical analysis, slides were deparaffinized in xylene and rehydrated sequentially in ethanol to water then incubated in 3% hydrogen peroxide to block endogenous peroxidase activity. Antigen retrieval was performed in 10 mM citrate buffer pH 6.0 (DAKO Cytomation, Carpinteria, CA) for 15 minutes in a microwave oven. Nonspecific antibody binding was blocked by incubating slides with Biocare Blocking Reagent (Biocare, Concord, CA) for 10 minutes. Slides were washed and then incubated at 4°C with primary antibody: Ki-67 (1∶200 overnight at 4°C; DAKO Cytomation, Carpinteria, CA), p53 (1∶500 for 1 hour at room temperature; Navocastra Laboratories Ltd, Newcastle, UK), ERα (1∶500 for 1 hour at room temperature; Santa Cruz Biotechnology, Santa Cruz, CA), E-cadherin (1∶50 for 1 hour at room temperature; Santa Cruz Technology), slug (1∶50 overnight, Santa Cruz Technology) and p21 (1∶50 overnight; Santa Cruz Biotechnology). Finally, slides were incubated with Dako EnVision™ labeled polymer for 30 minutes at room temperature, followed by incubation with Dako diaminobenzidine and counterstained with hematoxylin. Tumor slides were scanned using the ScanScope XT (Aperio Technologies, Vista, CA). Quantitation was performed using the Aperio Digital Pathology Platform. Briefly, 3–4 representative areas/tumor (n = 5 tumor sections per diet and genotype group) were viewed at 20× magnification and scored based on stain intensity (for E-cadherin) or the percentage of cells with positive staining (for all other markers). Positive staining for ERα and slug was further stratified by 1+, low; 2+, moderate; and 3+, intense staining. Average scores for each stain were calculated by 3 experienced blinded reviewers and the results were within strong agreement.

### DNA Damage and Western Blot

Wnt-1 p53^+/+^ or Wnt-1 p53^+/−^ mammary cell suspensions were exposed to 300 J/m^2^ of UVC and allowed to incubate in RPMI-160 media for 0, 6 or 24 hours. Centrifuged cell pellets were lysed in radioimmunoprecipitation assay buffer (Sigma, St. Louis, MO) with protease inhibitor tablet (Roche Applied Sciences, Indianapolis, IN) and phosphatase inhibitor cocktails I and II (Sigma). Protein lysates (40 µg) were resolved by SDS-PAGE using 12% gels, transferred to nitrocellulose and blocked using LI-COR Blocking Buffer (LI-COR Biotechnologies, Lincoln, NE). Membranes were incubated overnight at 4°C with primary antibody (Cell Signaling, Boston, MA; 1∶1000). After 3 washes (5 minutes each) in 0.1% Tween-20/PBS (PBS-T), membranes were incubated for 1 hour at RT in species-specific secondary antibody (LI-COR Biotechnologies) diluted in LI-COR blocking buffer (1∶5,000). Following 3 washes in PBS-T, membranes were scanned using the Odyssey infrared fluorescent imaging system. Densitometry was performed using LI-COR Software (LI-COR Biotechnologies).

### Quantitative Real-time PCR

RNA was extracted from Wnt-1 tumor cell suspensions (approximately 1 million cells) or tumors (5 per diet and genotype groups) using the Trizol method (Invitrogen, Carlsbad, CA) per manufacturer’s instructions. RNA concentration was spectrophoretically determined using a NanoDrop (Thermo Scientific, Logan, UT) and RNA quality was confirmed by an Agilent 2100 Bioanalyzer (Agilent, Clara, CA). cDNA was synthesized using a reverse transciptase kit (Applied Biosciences, Austin, TX). Real time PCR was performed using the Taqman primer/probe system (Applied Biosciences) for coding RNA, and Exiqon (Woburn, MA) primers with SYBR technology (Applied Biosciences) for noncoding RNA. The reaction was monitored by a ViiATM7 Real time PCR system (Applied Biosciences). Data are presented as gene expression relative to that in Wnt-1 p53^+/+^ tumors from control mice.

### Statistics

All statistical analyses were conducted by Student’s t-test using SAS 9.3 (Cary, NC). Data are presented as mean ± SD. Data not meeting assumptions of normality were transformed by natural log; this included the data on serum insulin and leptin, p21 expression in Wnt-1 p53^+/−^ tumors, and miR125-b expression in both tumor genotypes. Differences were considered significant at P≤0.05.
